# Amyloid Oligomer Conformation in a Group of Natively Folded Proteins

**DOI:** 10.1371/journal.pone.0003235

**Published:** 2008-09-18

**Authors:** Yuji Yoshiike, Ryoichi Minai, Yo Matsuo, Yun-Ru Chen, Tetsuya Kimura, Akihiko Takashima

**Affiliations:** 1 Laboratory for Alzheimer's Disease, RIKEN Brain Science Institute, Wako-shi, Saitama, Japan; 2 Computational Proteomics Team, RIKEN Genomics Sciences Center, Tsurumi-ku, Yokohama, Japan; 3 The Genomics Research Center, Academia Sinica, Taipei, Taiwan; University of Washington, United States of America

## Abstract

Recent in vitro and in vivo studies suggest that destabilized proteins with defective folding induce aggregation and toxicity in protein-misfolding diseases. One such unstable protein state is called amyloid oligomer, a precursor of fully aggregated forms of amyloid. Detection of various amyloid oligomers with A11, an anti-amyloid oligomer conformation-specific antibody, revealed that the amyloid oligomer represents a generic conformation and suggested that toxic β-aggregation processes possess a common mechanism. By using A11 antibody as a probe in combination with mass spectrometric analysis, we identified GroEL in bacterial lysates as a protein that may potentially have an amyloid oligomer conformation. Surprisingly, A11 reacted not only with purified GroEL but also with several purified heat shock proteins, including human Hsp27, 40, 70, 90; yeast Hsp104; and bovine Hsc70. The native folds of A11-reactive proteins in purified samples were characterized by their anti-β-aggregation activity in terms of both functionality and in contrast to the β-aggregation promoting activity of misfolded pathogenic amyloid oligomers. The conformation-dependent binding of A11 with natively folded Hsp27 was supported by the concurrent loss of A11 reactivity and anti-β-aggregation activity of heat-treated Hsp27 samples. Moreover, we observed consistent anti-β-aggregation activity not only by chaperones containing an amyloid oligomer conformation but also by several A11-immunoreactive non-chaperone proteins. From these results, we suggest that the amyloid oligomer conformation is present in a group of natively folded proteins. The inhibitory effects of A11 antibody on both GroEL/ES-assisted luciferase refolding and Hsp70-mediated decelerated nucleation of Aβ aggregation suggested that the A11-binding sites on these chaperones might be functionally important. Finally, we employed a computational approach to uncover possible A11-binding sites on these targets. Since the β-sheet edge was a common structural motif having the most similar physicochemical properties in the A11-reactive proteins we analyzed, we propose that the β-sheet edge in some natively folded amyloid oligomers is designed positively to prevent β aggregation.

## Introduction

Protein misfolding diseases are characterized by the formation of amyloid, which occurs through misfolding promoted by the conversion of a protein from its native to non-native state. Under the appropriate conditions, any proteins can form generic amyloid [1]. The ability of an oligomeric entity in amyloid to seed the polymerization of other proteins indicates that amyloid may disrupt cellular functions by interfering with the folding of other proteins [2]–[5, Kayed et al., *Society for Neuroscience Abstracts* 2005;35:893.6]. Therefore, it is important to understand the molecular mechanism(s) underlying the seeding function of amyloid oligomers, whose pathogenic significance in protein misfolding diseases have been well supported. Because amyloid oligomers innately tend to aggregate, high-resolution elucidation of their structures through conventional physical techniques has been challenging. Novel insight on the structure of amyloid oligomers was made possible by the development of an anti-amyloid oligomer conformation-dependent antibody, A11 [2]. The fact that various amyloid oligomers are A11 immunopositive suggests that amyloid oligomers share a common structure and implies that various protein-misfolding diseases may have a common pathogenic mechanism [2]. In the present study, we identified proteins that contain the amyloid oligomer conformation by using the A11 antibody as a probe. One group of A11-reactive proteins commonly displayed anti-β-aggregation activity as opposed to the expected β-aggregation promoting activity of misfolded amyloid oligomers.

## Results

### Amyloid oligomer conformation in chaperones

**Figure 1:**
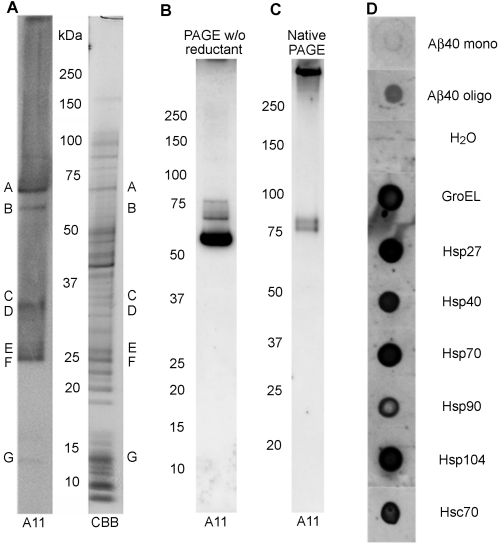
Amyloid oligomer conformation in chaperones. (A) *E. coli* competent cells (strain DH5α; TOYOBO, Japan) were freeze-thawed three times by freezing the cells on ice at −180°C then thawing them at 37°C. The lysates were then mixed with sample buffer lacking SDS and reducing agents and then subjected to PAGE analysis. One gel was immunoblotted with A11 antibody. A11-immunoreactive bands (*A–G*) were excised from the CBB-stained gel, treated with trypsin, and analyzed by tandem-mass spectrometry. A candidate A11-reactive protein from band *B* was GroEL. The difference in staining patterns between the A11 immunoblot and CBB-stained gel indicates A11 antibody-binding selectivity. Note also that the proteins in cell lysates reacted with the A11 antibody are not necessarily all natively folded. Purified GroEL (23 µM) was mixed with sample buffer lacking both reducing agents and SDS, and then run on (B) a gel in a regular SDS-containing buffer or (C) a gel in a buffer lacking SDS (native PAGE). Gels were blotted onto a membrane that was subsequently probed with A11 under the same conditions. Note that the mobility of the molecular weight standard is different in (B) and (C). (D) The following solutions (3 µL each) were dot blotted onto a nitrocellulose membrane: 20 µM Aβ40 monomer, 20 µM Aβ40 oligomer, H_2_O, purified 23 µM GroEL, 37 µM Hsp27, 20 µM Hsp40, 14 µM Hsp70, 12 µM Hsp90, 10 µM Hsp104, and 10 µM Hsc70. GroEL, Hsp27, Hsp40, Hsp90, and Hsc70 were purchased from Stressgen (Canada). Hsp70 was from Sigma (USA). Hsp104 was from ATGen (South Korea). Each protein solution was blotted onto a single membrane that was probed with A11 under the same conditions. The individual dot blots were arranged into a single column.

A11 is an antibody that specifically detects the conformation of amyloid oligomers regardless of their amino acid sequence [2]. To determine whether proteins that contain the amyloid oligomer conformation exist in bacteria, we examined the A11 immunoreactivity of *Escherichia coli* cell lysates. We chose to examine bacterial proteins because extensive structural information about these proteins already exists in the protein data bank (PDB). We subjected *E. coli* DH5α cell lysates to PAGE analysis. To reduce false-positive signals, we used sample buffer that contained neither SDS nor reducing agents to solubilize the lysates although the disruption of conformation was predicted to some extent during SDS-PAGE. One gel was Western blotted with A11 to identify the molecular weights of A11-immunoreactive proteins (Figure 1A), and another gel was stained with coomassie brilliant blue (CBB). The bands in the CBB-stained gel corresponding to the A11-immunoreactive bands on the immunoblot were analyzed by tandem mass spectrometry (MS-MS). We identified one of these A11-immunoreactive bands to contain GroEL, a bacterial chaperonin [6]. Unlike other proteomics studies that have used sequence-dependent antibodies, in our study, the proteins detected in the cell lysates through this simple approach remain unconfirmed candidates of A11-reactive proteins. Indeed, the conformational specificity of A11 for each protein (purified and native forms) needs to be analyzed, since the cell lysis procedure might have affected protein conformation to unknown degrees.

To confirm the A11 immunoreactivity of GroEL, we performed Western analysis on purified GroEL and observed that A11 strongly immunostained monomeric GroEL (Figure 1B). Although the sample buffer used in this PAGE analysis lacked SDS and reducing agents, the running buffer did contain SDS. In true native PAGE, which is performed without SDS, A11 mostly immunostained a high molecular weight band representing a GroEL oligomer complex, which is the expected functionally active form of GroEL in bacterial cytosol (Figure 1C). A11 antibody also strongly immunostained purified GroEL on dot blots (Figure 1D). This enabled us to exclude the possibility that the PAGE processing misfolded GroEL molecules that potentially could react with A11 and lead to false A11 positives. A11 antibody also immunostained purified recombinant human chaperones, including heat shock protein (Hsp) 27, 40, 70, 90; yeast Hsp104; and bovine heat shock cognate (Hsc) 70, albeit with varying intensity (Figure 1D). As expected, A11 antibody immunostained β-amyloid (Aβ) oligomer prepared from Aβ(1–40) but not monomeric Aβ. These results suggest that certain chaperone structures share a common amyloid oligomer conformation. Among the A11-reactive candidates, we were interested in identifying chaperones that normally display anti-aggregation activity as opposed to proteins, such as misfolded pathogenic amyloid oligomers, that induce β-aggregation of other proteins [2]–[5, Kayed et al., *Society for Neuroscience Abstracts* 2005;35:893.6].

### Proteins containing the amyloid oligomer conformation affect Aβ aggregation in opposite ways

**Figure 2:**
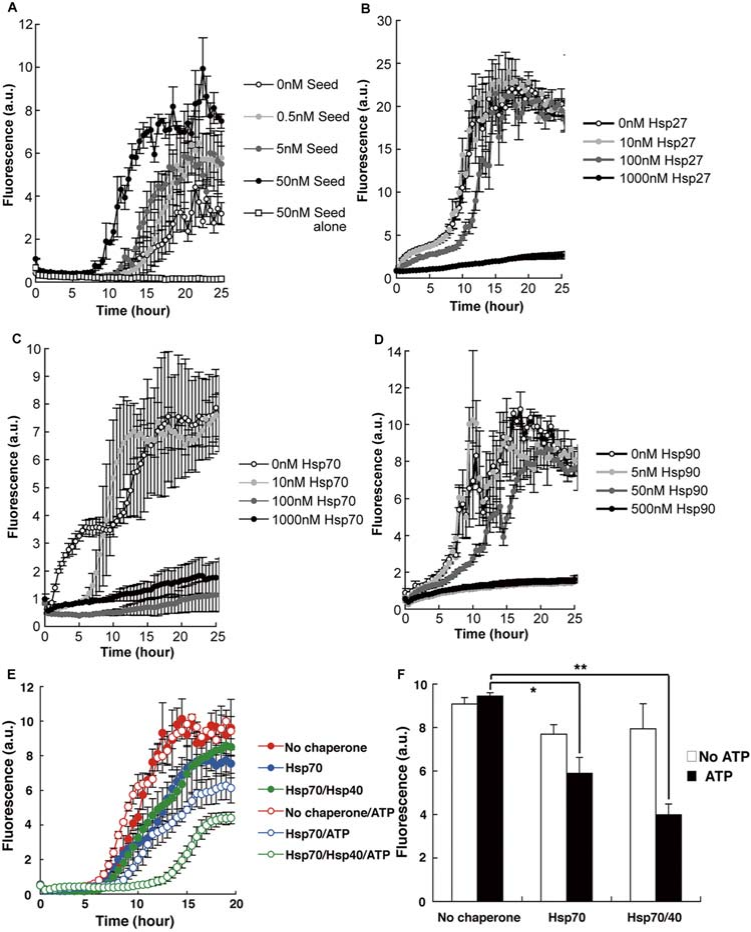
Contrasting effects of Aβ oligomer and chaperones on Aβ aggregation. (A) Aβ40 (final concentration: 5 µM) dissolved in aCSF was mixed with 0, 0.5, 5, or 50 nM Aβ40 oligomer (i.e., “seed”) in H_2_O. Results from a sample containing only 50 nM Aβ40 oligomer is also shown. ThT fluorescence intensity indicates the level of Aβ aggregation. (B) Aβ40 (final concentration: 10 µM) dissolved in H_2_O was mixed with 0, 10, 100, or 1000 nM Hsp27 in buffer (1.08 mM Tris-HCl [pH 7.5], 541 µM NaCl, 54.1 µM EDTA, and 54.1 µM DTT). (C) Aβ40 (final concentration: 10 µM) dissolved in H_2_O was mixed with 0, 10, 100, or 1000 nM Hsp70 in buffer (2.7 mM KCl, 1.5 mM KH_2_PO_4_, 137 mM NaCl, and 8.1 mM Na_2_HPO_4_). (D) Aβ40 (final concentration: 5 µM) dissolved in H_2_O was mixed with 0, 5, 50, or 500 nM Hsp90 in buffer (3.9 mM Tris-HCl [pH 7.5], 7.8 mM NaCl, and 78.2 µM DTT). (E) Anti-β-aggregation activity of Hsp40 and Hsp70. Aβ40 (5 µM) dissolved in aCSF was mixed with either 10 nM Hsp70 in buffer A (15.1 µM KCl, 8.4 µM KH_2_PO_4_, 767 µM NaCl, and 45.3 µM Na_2_HPO_4_) or a mixture of 10 nM Hsp70 in Buffer A and 10 nM Hsp40 in Buffer B (10.8 µM KCl, 6.0 µM KH_2_PO_4_, 548 µM NaCl, and 32.4 µM Na_2_HPO_4_) in the presence or absence of 500 µM Mg-ATP. (F) Bar graph showing ThT fluorescence intensity of Aβ samples with or without chaperones after 17 h of incubation. Statistical significance was calculated by using a Student's t-test. *p = 0.035; **p = 0.00046. Data are presented as means+/−SEM.

To confirm the seeding property of pathogenic Aβ oligomer, we assessed the β-aggregation kinetics of Aβ40 in the presence of preformed Aβ oligomer using a thioflavin T (ThT) fluorescence assay [7] (Figure 2A), and found that Aβ oligomer induced Aβ aggregation in a dose-dependent manner. To determine whether the chaperones we assessed with A11 antibody were functionally active and thus natively folded or misfolded like pathogenic amyloid oligomers, we examined the effects of Hsp27, Hsp70, and Hsp90 on Aβ-aggregation kinetics using the ThT assay [7] (Figure 2B–D). Aβ, a type of amyloid protein observed in Alzheimer's disease (AD), has been pathologically linked to these three chaperones [8]. As with other published studies [9], [10], we found that all three chaperones—Hsp27, Hsp70, and Hsp90—suppressed the β aggregation of Aβ in a dose-dependent manner to varying degrees. We did not observe fibrillar amyloid structures in Aβ samples incubated with each of these chaperones under an electron microscope (data not shown). To verify this chaperone activity [6], we examined whether the anti-β-aggregation properties of Hsp was energy dependent (Figure 2E, F). Adding ATP to samples containing Hsp70 or Hsp70/Hsp40 at concentrations that alone do not show anti-β-aggregation activity caused Hsp70 and Hsp70/Hsp40 to significantly suppress Aβ aggregation. Most of the purified recombinant chaperones used here, including Hsp70 and Hsp40, have tested positive for their ATPase activity. The energy-dependent anti-β-aggregation activity of these chaperone samples indicates that A11-immunoreactive chaperones are indeed functional; thus, a significant number of chaperone molecules in these purified samples are natively folded [4], [11]. In other words, the native structures of these chaperones possess the amyloid oligomer conformation. Therefore, there are at least two classes of A11-reactive proteins that contain the amyloid oligomer conformation: pathogenic amyloid oligomers that promote β aggregation and chaperones that suppress β aggregation.

### Heating affects the amyloid oligomer conformation and anti-β-aggregation activity of Hsp27

**Figure 3:**
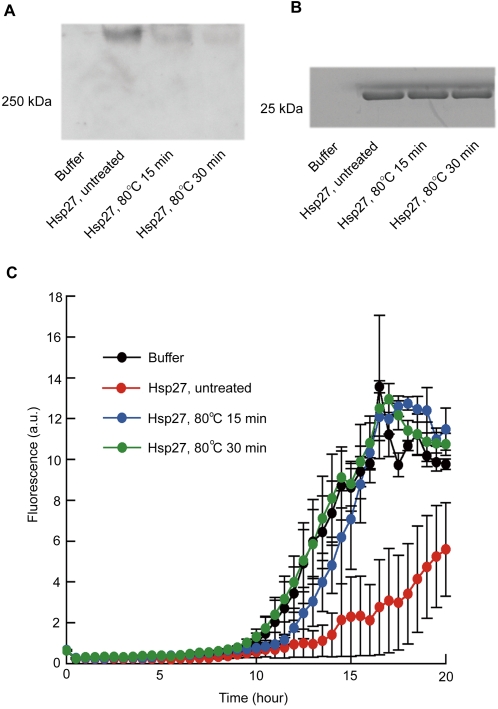
Effects of heat treatment on amyloid oligomer conformation in Hsp27 and the anti-β-aggregation activity of Hsp27. (A) Western blot of Hsp27 probed with A11 anti-amyloid oligomer conformation-dependent antibody. Hsp27 samples were heat treated at 80°C for 15 or 30 min. Heat-treated and untreated Hsp27 samples (10 µM) were mixed with sample buffer lacking SDS and reducing reagents, subjected to native PAGE using 4–20% gels, and separated proteins were immunoblotted with A11 antibody. Note that A11-immunoreactive Hsp27 bands migrated to a high molecular-weight level in native PAGE, because Hsp27 exists as large oligomers having chaperone-like activity [8], [12]. (B) Heat-treated and untreated Hsp27 samples (10 µM) were mixed with sample buffer containing SDS and β-mercaptoethanol, and then subjected to SDS-PAGE using 4–20% gels. Gels were stained with coomassie brilliant blue (CBB). (C) Aβ-aggregation kinetics of samples containing heat-treated Hsp27. One buffer sample and three Hsp27 samples were diluted 100 times with H_2_O. Aβ40 (final concentration: 5 µM) dissolved in aCSF was mixed with buffer (54 µM Tris-HCl [pH 7.5], 27 µM NaCl, 2.7 µM EDTA, and 2.7 µM DTT); untreated 50 nM Hsp27; 15-min heat-treated Hsp27; or 30-min heat-treated Hsp27. All samples were incubated in the presence of 10 µM ThT; triplicate measurements were taken every 30 min at excitation and emission wavelengths of 440 nm and 486 nm [7], respectively. Data are presented as means+/−SEM.

Next, we examined how heat treatment alters the amyloid oligomer conformation of Hsp27 and the Hsp27-mediated suppression of Aβ aggregation (Figure 3). We assessed Hsp27 because of its strong A11 immunoreactivity on both dot blot and native-PAGE/Western blot analyses, and because of its pathological association with Aβ [8]. We heated Hsp27 dissolved in buffer to 80°C for 15 and 30 min and then subjected the sample to native-PAGE followed by Western blotting with A11 antibody. Heat treatment abolished A11 immunoreactivity of Hsp27 (Figure 3A), even though the peptide backbone of Hsp27 was intact (SDS-PAGE of heat-treated samples, Figure 3B). ThT assay of samples containing Aβ and heat-treated Hsp27, however, showed that Hsp27-mediated suppression of Aβ aggregation disappeared concurrently with the reduction of amyloid oligomer conformation resulting from the heat treatment (Figure 3C). The heat-denaturing experiments simply suggested that the conformation-specific binding of A11, and thus the amyloid oligomer conformation—not non-specific interactions—was consistently observed as natively folded Hsp27 prevented β aggregation. These results certainly did not demonstrate that the A11-reactive domain within Hsp27 is responsible for suppressing Aβ aggregation. Both the structure and functional mechanisms of Hsp27 are unknown. Since the spherical and oligomeric forms of Hsp27 are active states [12], Hsp27 could co-oligomerize with Aβ while suppressing amyloidogenesis. Heat-treated Hsp27 may have also formed amyloid that complexed with Aβ. Taken together, these data identify the amyloid conformation as the generic structural feature of natively folded chaperones directly correlating with the anti-β-aggregation activity of chaperones.

### A11 immunoreactivity and anti-β-aggregation activity in non-chaperone proteins

**Figure 4:**
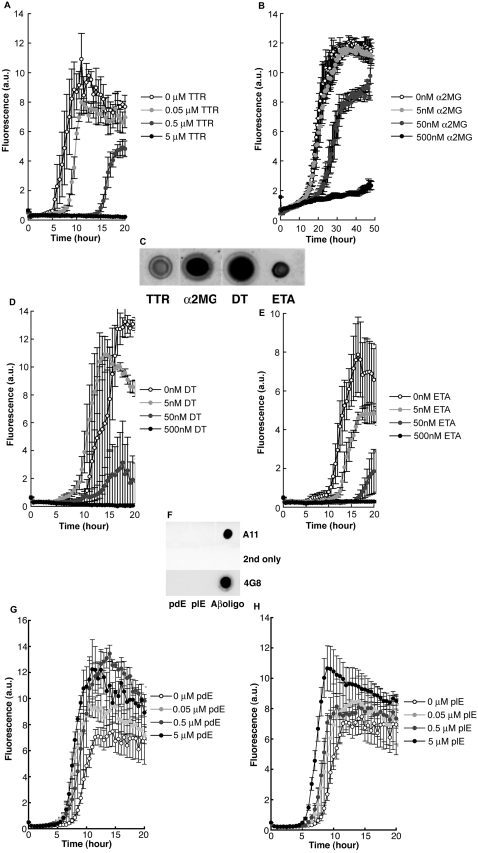
Dose-dependent suppression of Aβ aggregation by A11-immunoreactive non-chaperone proteins. (A) Aβ40 (final concentration: 5 µM) dissolved in aCSF was mixed with 0, 50, 500, or 5000 nM TTR in 10 mM Tris-HCl (pH 7.4) with 0.9% NaCl. ThT fluorescence indicates the level of Aβ aggregation. (B) Aβ40 (final concentration: 5 µM) dissolved in H_2_O was mixed with 0, 5, 50, or 500 nM α2MG in buffer (1.45 mM Tris-HCl [pH 8.0], 9.4 mM glycine, and 5.8 mM trehalose). (C) Dot blot of 100 µM TTR, 13.8 µM α2MG, 17.2 µM DT, and 15.2 µM ETA (3 µl each) probed with A11 antibody [2]. (D) and (E) Aβ40 (final concentration: 5 µM) dissolved in aCSF was mixed with buffer alone; 5, 50, or 500 nM DT; or ETA. For (D), the buffer contained 10 mM Tris-HCl (pH 7.4) and 1 mM EDTA. For (E), the buffer contained 10 mM sodium phosphate (pH 7.5) and 150 mM NaCl. (F) Dot blot of 100 µM pdE (approx. MW: 50 kDa); 10 mM plE (approx. MW: 1000 Da); and 50 µM Aβ40 oligomer (3 µl each) probed with A11, secondary antibody alone without primary antibody, and 4G8 antibody (which reacts to amino acid residues 17–24 of human Aβ). (G) and (H) Aβ40 (final concentration: 5 µM) dissolved in PBS was mixed with buffer alone; 0.05, 0.5, 5 µM pdE; or plE. Data are presented as means+/−SEM.

Anti-β-aggregation activity is not unique to chaperones. Thus, if chaperones with anti-β-aggregation activity contain the amyloid oligomer conformation, we assumed that other proteins known to suppress Aβ aggregation might also contain the amyloid oligomer conformation. One such protein is transthyretin (TTR), which has been shown to sequester Aβ and prevent amyloid formation [13], [14]. We confirmed the suppressive effect of TTR on Aβ aggregation in our model system (Figure 4A). Another protein with anti-Aβ-aggregation properties is α2-macroglobulin (α2MG). Although the genetic association between α2MG and AD remains controversial [15], the anti-β-aggregation activity of α2MG against Aβ has been reported to occur *in vitro* [16], [17], as we also observed (Figure 4B). As expected, A11 antibody immunostained dot blots of TTR and α2MG (Figure 4C), indicating that both proteins possess the amyloid oligomer conformation. Although TTR is a protein that forms amyloid in pathogenic conditions, its A11 immunoreactivity has never been reported [18]. If a significant number of TTR molecules somehow misfold and bind to A11 in solution, they are more likely to induce or cross-seed Aβ aggregation, as other misfolded pathogenic amyloid oligomers [2]–[5, Kayed et al., *Society for Neuroscience Abstracts* 2005;35:893.6]. In our experiment, this did not occur. Instead TTR suppressed Aβ aggregation (Figure 4A) [13], [14], indicating that the amyloid oligomer conformation within TTR is not misfolded but is natively folded.

Analysis of the crystal structures of the TTR and α2MG receptor-binding domains reveal that both contain a β sandwich with a jelly-roll topology [19], [20]. The crystal structures of diphtheria toxin (DT) and exotoxin A (ETA) also have a homologous jelly-roll-like topology [21], [22]. As expected, A11 antibody also immunostained both DT and ETA (Figure 4C). Surprisingly, these amyloid oligomer conformation-containing toxins also suppressed Aβ aggregation *in vitro* in a dose-dependent manner, even though they have no known physiological relationship to Aβ (Figure 4D, E). These results suggest that A11 immunoreactivity is not only found in chaperones but is also found among certain non-chaperone proteins possessing anti-β-aggregation activity.

Comparative analyses require control experiments. Thus in the present study, we tested whether A11-immunonegative proteins fail to inhibit β aggregation. Different chain lengths of D-poly glutamic acids (pdE) and L-poly glutamic acids (plE) did not exhibit A11 immunoreactivity or inhibit β aggregation, instead they promoted β aggregation (Figure 4F–H). These results support the conformational specificity of A11 antibody binding. Indeed, plE mostly exists as a random coil at neutral pH [23], the same pH we used in our control experiment. These results also suggest that A11 reactivity, and thus amyloid oligomer conformation, is not the only functional determinant of β-aggregation-promoting activity. Because we did not test the anti-β-aggregation activity of all purified and folded A11-non-reactive proteins, we could not exclude the possibility that an A11-non-reactive protein possessing anti-β-aggregation activity exists. For the same reason, our current results cannot exclude the possible existence of A11-reactive folded proteins that do not possess anti-β-aggregation activity. Therefore, we do not advance the idea that A11 immunoreactivity is a prerequisite for anti-β-aggregation activity.

### A11 affects GroE-assisted luciferase refolding and Hsp70-decelerated Aβ nucleation

**Figure 5:**
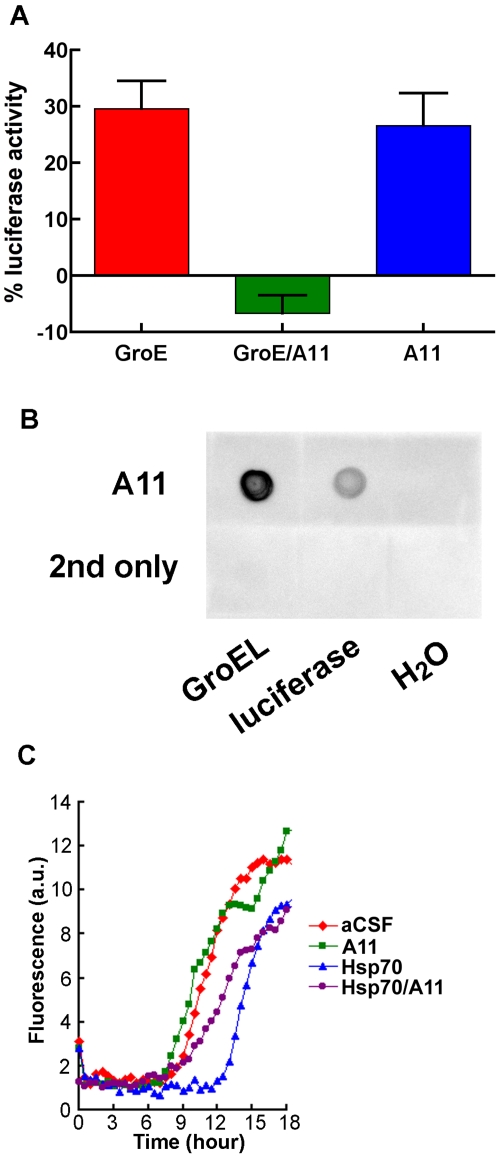
A11-mediated inhibition of GroE-assisted luciferase refolding and Hsp70-mediated decelerated Aβ nucleation. (A) In a sample of GroE (final concentrations: 1.25 µM GroEL and 2.1 µM GroES) and luciferase (final concentration: 100 nM), about 30% of luciferase activity was recorded relative to the activity of luciferase samples without heat-treatment, indicating that GroE-assisted partial refolding of heat-denatured luciferase had occurred. Luciferase activity was not observed when heat-treated luciferase was co-incubated with both GroE and A11 (final concentration: 0.1 mg/mL). A11 antibody assisted the refolding of luciferase (n = 15 for GroE and n = 6 for GroE/A11 and A11 alone, respectively). Data are presented as means+/−SEM. (B) Three microliters of 250 nM GroEL, 2.5 µM luciferase, and H_2_O were blotted onto a nitrocellulose membrane. The membrane was incubated either with the A11 antibody in 5% skim milk or with just skim milk. (C) Aβ40 (final concentration: 5 µM) dissolved in aCSF was mixed with or without Hsp70 (final concentration: 75 nM) and A11 (final concentration: 300 nM). All samples were incubated in the presence of 5 µM ThT. Triplicate measurements were taken every 30 min. Average fluorescence values are plotted. Error bars were omitted from the graph for clarity.

To determine whether the A11-binding site plays a functional role in protein folding, we first investigated the effects of A11 antibody on GroEL/ES (GroE)-assisted firefly luciferase refolding. Luciferase samples were heated at 45°C for 5 min. After cooling the samples at room temperature for 5 min and adding luciferin substrate, GroEL/ES and ATP facilitated luciferase refolding, which was evident by the increased luminescence in the samples (Figure 5A). Luminescence was not observed in heat-treated samples co-incubated with GroEL/ES and ATP in the presence of A11. This suggests that A11 prevented GroEL/ES-assisted refolding of heat-denatured luciferase. Surprisingly, A11 itself assisted luciferase refolding to a similar extent as GroEL/ES.

Antibody-assisted refolding of proteins, including luciferase, has been reported previously in cases in which an antibody specific to a given protein facilitates antigen folding [24], [25]. Therefore, we surmised that the A11-assisted refolding of luciferase we observed might involve A11 binding to luciferase, which also possesses a β motif [26]. Thus, we tested the A11 immunoreactivity of purified luciferase by dot blot (Figure 5B). The luciferase sample exhibited A11 immunoreactivity that was weaker than that of the positive GroEL control blotted on the same membrane. These results provide a possible explanation for the assisted refolding of luciferase by A11: Like other antibodies having chaperone-like activity, A11 may induce luciferase refolding through specific interactions. If this is the case, then the diminished refolding of luciferase in samples containing both chaperonin and A11 is more likely due to the interaction of A11 with chaperonin rather than the interaction of A11 with luciferase.

Next, we examined how A11 affects Hsp70-mediated suppression of Aβ aggregation by assessing Aβ aggregation kinetics (Figure 5C). The presence of Hsp70 decelerated the nucleation of Aβ aggregation. This effect was suppressed by A11, even though elongation was not substantially affected. This result indicates that the A11-binding site on Hsp70 may play a role in inhibiting nucleation but not elongation during Aβ aggregation. Interestingly, A11 alone failed to substantially affect Aβ aggregation at the concentration used. It will be interesting to examine in further detail whether A11 assists or prevents the oligomerization of Aβ and other amyloidogenic proteins. The present results indicate that A11-binding sites on chaperones may be functionally important.

### Deduction of A11-epitope and amyloid oligomer conformation through structural similarity search

**Figure 6:**
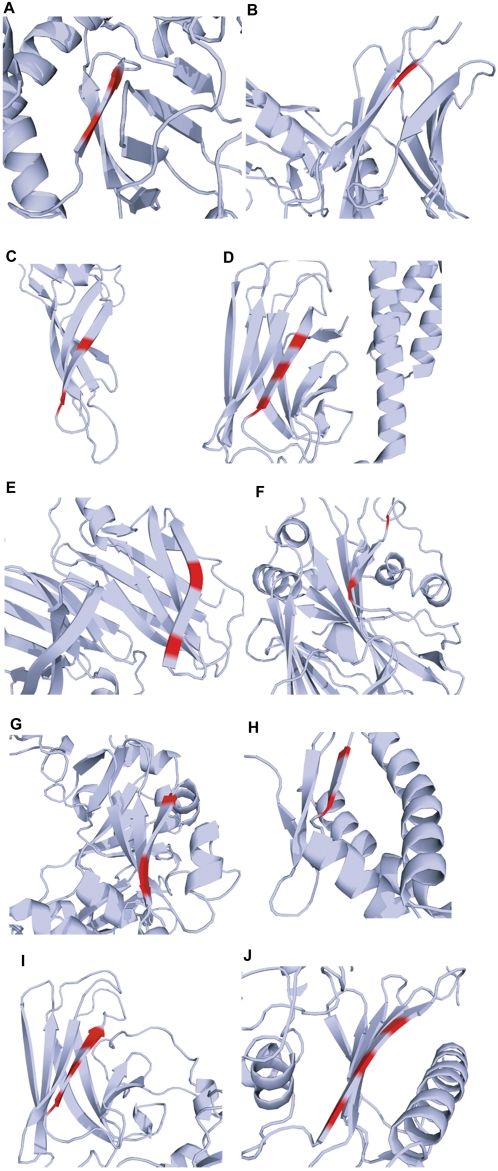
Sites with physicochemical properties of greatest similarity among ten A11-reactive proteins. The crystal structures of ten A11-reactive proteins were aligned to identify the areas where the physicochemical properties of solvent-exposed atoms show the highest similarity (red). (A) GroEL [1aon]. (B) α2MG [1ayo]. (C) Hsp40 [1c3g]. (D) DnaK (Hsp70) [1dkz]. (E) TTR (prealbumin) [1dvq]. (F) ETA [1ikq]. (G) DT [1mdt]. (H) ClpB (Hsp104) [1qvr]. (I) Hsc70 [1yuw]. (J) Hsp90 [2cg9]. The PDB codes of the structures used in the structural similarity search are shown in square brackets.

Our analysis of A11 immunoreactivity and anti-β-aggregation activity of purified proteins led us to identify at least ten A11-reactive folded proteins with reported crystal structures. These included associated protein fragments and corresponding homologues from different species. Information about A11-immunoreactive proteins and their atomic structures may help us deduce the conformation shared by amyloid oligomers. To determine the A11 epitope and thus amyloid oligomer conformation, we searched each of the ten crystal structures for areas sharing the most surface physicochemical properties (see Methods). For the similarity search, we placed two main restrictions according to the known properties of amyloid oligomer conformations. First, the search was limited to only structures having a β strand, which is a generally accepted basic component of amyloid. Second, the search was limited to only areas that are less influenced by amino acid side chains, because A11 immunoreactivity is conformation dependent not amino-acid-sequence dependent [2]. Areas showing the most similarity are shown in red for each of the ten crystal structures (Figure 6 and Figure S1). All areas were located at the side edge of the β-sheet conformation, which comprises most of larger β-structural motifs such as the β-sandwich motif. Some of the potential A11-binding areas were located at or near the suggested substrate-binding domain of chaperones (e.g., GroEL, Hsp70, Hsc70, Hsp40) [27]–[30], while other potential A11-binding areas were not (e.g., ClpB [Hsp104] and Hsp90) [31]–[33]. The Aβ-binding site of TTR, previously identified through site-directed mutagenesis as well as through molecular modeling [13], [34], partially matched the area proposed here as the A11 epitope. Interestingly, the displacement of this particular terminal β strand by a conformational change promotes fibril formation of TTR itself [35]. These observations support the hypothesis that the β-sheet edge, a common feature of the ten crystal structures we assessed, plays a key role in A11-reactive proteins. Indeed, the negative design of the β-sheet edge supposedly helps natural β-sheet proteins to avoid β aggregation [36]. Here, we propose that β-sheet edge in natively folded amyloid oligomers not only protects proteins from undergoing β aggregation but also prevents interacting molecules from undergoing β aggregation. Thus, in some cases this can be considered to be a positive design. How the amyloid oligomer conformation influences protein conformation still needs to be determined in the future. This can be achieved by investigating the detailed molecular interactions between chaperone/chaperone-like proteins and the A11 antibody and between these proteins and amyloidogenic proteins.

## Discussion

By using the A11 antibody, an antibody known to detect the conformation of amyloid oligomers [2], we unexpectedly observed the amyloid oligomer conformation in a group of proteins whose folds were functionally characterized as native on the basis of their anti-β-aggregation activity. This contrasts with the misfolded pathogenic amyloid oligomers that promote β aggregation [2]–[5, Kayed et al., *Society for Neuroscience Abstracts* 2005;35:893.6]. Because we propose a novel corollary to the original claim that A11 recognizes a common structure shared by oligomeric intermediates formed by a diverse range of amyloidogenic proteins [2], our results were carefully examined.

Could the recognition by A11 of the identified proteins be a non-specific effect caused, for example, by a small fraction of partially misfolded A11 antibody? We considered that this was unlikely because heat-denatured Hsp27 did not bind A11 (Figure 3). A11 reacted with some non-chaperone proteins (Figure 4). Moreover, none of the identified A11-immunoreactive proteins bound secondary antibody in samples lacking A11 (data not shown).

Could the anti-β-aggregation activity of the identified proteins be induced by colloidal or crowding effects? We considered this to be unlikely because Hsp70/40 exhibited energy-dependent anti-β-aggregation activity (Figure 2E). Heat-treated Hsp27 (Figure 3C) and polyglutamate (Figure 4G,H) did not show the anti-β-aggregation activity. In addition, crowded GroE/A11 mixtures did not facilitate luciferase refolding more than less crowded GroE or A11 solutions (Figure 5A). Along similar lines, crowded Hsp70/A11 mixtures did not enhance anti-β-aggregation activity more than less crowded Hsp70 solutions (Figure 5C).

Could A11 be recognizing traces of misfolded amyloidogenic intermediates of these proteins? We considered that this was unlikely because if the contaminating molecules of such misfolded amyloid intermediates were substantially present in the sample, then we would expect the A11-reactive proteins, especially non-chaperone proteins like toxins, not to suppress β aggregation (Figure 4D, E). Rather, we would expect them to promote β aggregation as Aβ oligomers did (Figure 2A), because misfolded amyloid oligomers characteristically promote oligomerization and fibrillization of other proteins, a suggested pathogenic mechanism underlying protein misfolding diseases [2]–[5, Kayed et al., *Society for Neuroscience Abstracts* 2005;35:893.6]. A functional property that was commonly observed in the group of A11-reactive proteins was anti-β-aggregation activity, which differentiated these proteins from misfolded pathogenic amyloid oligomers, which promote β-aggregation. Together with the conformation-specific nature of A11 reactivity, this phenomenon led us to conclude that a significant number of molecules in the purified samples were neither misfolded nor unfolded, but were most likely natively folded.

Does the notion of a “common structure” mean anything if it so diverse it encompasses the folded and misfolded proteins? Under the right conditions, any protein can form amyloid [1]. The formation of amyloid structures by poly amino acids supports the premise that amyloid formation occurs independent of specific side-chain contacts or amino acid sequences [37]. Although plE forms amyloid at pH 4.1, it exists mostly as a random coil at physiological pH [23]. Indeed, plE dissolved in a neutral pH buffer showed neither A11 immunoreactivity nor anti-β-aggregation activity, which is consistent with the conformational specificity of A11 binding (Figure 4F–H). The difference in the staining patterns in immunoblots probed with A11 and in CBB-stained gels of bacterial cell lysate samples (Figure 1A) indicates that A11 does not bind just any protein. Not all A11-reactive proteins in the bands were necessarily natively folded or retained their *in vivo* conformation, since lysing the cells can significantly affect the conformation of some proteins. We believe that A11 specificity does exist, as demonstrated in the original study [2]. It is most important to note, however, that the specificity of A11 is completely different from that of other antibodies that recognize specific amino acid sequences in that A11 reactivity depends on the three-dimensional conformation of a protein since A11 specifically binds oligomeric intermediates but not unfolded monomers or fibers of amyloidogenic proteins [2]. Thus, A11 reactivity is independent of an antigen's amino acid sequence, because A11 specifically binds oligomeric intermediates formed by a diverse range of amyloidogenic proteins. The conformational-dependent binding specificity of A11 also implies that the amyloid oligomer represents a generic conformation [2]. On the basis of this particular generic property of the A11 antibody, we believe that A11 antibody is also capable of binding a group of natively folded proteins, as we observed in the present study.

What is the functional significance of having an amyloid oligomer conformation within natively folded proteins? Function-blocking experiments clarified the functional importance of A11-binding sites on chaperones (Figure 5). To further tackle this issue structurally, we took a computational approach. A functional property shared by pathogenic amyloid oligomers and chaperones is that both affect the conformation of unfolded proteins. This property might depend on the structural plasticity that is shared by amyloid and the substrate-binding domain of chaperonin, called the apical domain [38]–[41]. The structural plasticity of amyloid is partially based on its polymorphism, the ability of one polypeptide to form aggregates of different structures [39]. Indeed, the polymorphic nature of amyloid sets it apart from other globular folded proteins with unique structures. Thus, it is unlikely that the amyloid oligomer conformation is represented by a unique structure.

On the other hand, amyloid also exhibits isomorphism, the ability of different polypeptides to display similar morphologies [2], [39]. Taking advantage of this property, we searched for areas containing similar physicochemical properties on the surfaces of the crystal structures of A11-reactive folded proteins (Figure 6 and Figure S1). Our analysis results were consistent with the proposed structures of amyloid protofilaments or precursors [42]–[46]. Although the non-native amyloid precursor of β2-microglobulin is highly native-like, its edge strands contain perturbations that normally protect β-sandwich proteins from self-association [43]–[46]. Structurally, the edges of a completely regular β-sandwich motif are inherently β-aggregation prone [36]. Coincidentally, our structural similarity search revealed that the β-sheet edge of GroEL was located in the apical domain. A pioneering study on GroEL suggested that chaperonins act by stabilizing non-native intermediates against off-pathway misfolding and aggregation [27]. Because β-sheet structures are primarily formed through main-chain hydrogen bonds [4], [6], the induction of β-strand formation in a substrate through the β-sheet edge in the apical domain of GroEL may normally function to prevent non-specific side-chain contacts and thus non-specific aggregation, but in certain cases promote β aggregation as reported previously [47], [48]. We consider this idea to be speculative but consistent with the formation of β-strands by non-native substrates in a complex structure with Hsp40, and consistent with Hsp40-dependent amyloid assembly in cells [30], [49].

Finally, we would like to mention evolutionary aspects of ‘native’ amyloid oligomers. The presence of the amyloid oligomer conformation within a natively folded protein indicates that the folding observed in this type of conformation is based on amino acid sequence, which is encoded in that particular protein's gene according to Anfinsen's hypothesis [11]. On the other hand, the unprecedented diversity of proteins containing amyloid oligomer conformations also indicates that the folding in these conformations may be caused by something else, especially in pathogenic conditions [1], [2], [4]. In evolution, aggregative properties of some proteins may have pressured the selection of anti-aggregation properties in some genes as a preventive measure. On the basis of the generic anti-aggregation characteristics of certain A11-reactive folded proteins, such as the chaperone family, we hypothesize that (1) the amyloid oligomer conformation has evolved over time as a means to prevent non-specific protein aggregations, and that (2) based on the structural commonality, amyloidogenic proteins may sporadically deviate from this system of preventing protein aggregation in some conditions such as aging in the extended life span of humanity today that has not been caught up by evolution.

## Materials and Methods

### Immunoblot (Western blot and dot blot)

Separated proteins by SDS-PAGE were transferred onto nitrocellulose membranes by using a wet system at 4°C. Alternatively, 3 µL of sample solution was dot-blotted onto membranes. After being blocked with milk, the blotted membrane was probed with anti-amyloid oligomer antibody A11 (1∶1000; Biosource, USA) [2] or 4G8 (1∶1000; Signet, USA). A11 and 4G8 antibody immunoreactivity were detected with HRP-conjugated anti-rabbit IgG (1∶2500) or anti-mouse IgG (1∶5000), respectively, followed by ECL. A replicated membrane probed with the secondary antibody alone did not display signals. Each panel displaying an immunoblot was from a single membrane and thus a single experiment. For presentation purposes, the squares containing each dot blot were arranged in a single column or row for figures.

### Aβ oligomer preparation

Aβ oligomer was prepared as described previously [2]. Briefly, lyophilized Aβ40 peptide (Peptide Institute, Japan) was dissolved in 1,1,1,3,3,3-hexafluoro-2-propanol (HFIP) (Wako, Japan) on ice and aliquoted to be frozen until use. Aβ was spin-vacuumed just prior to the experiment; dissolved in HFIP solution (final concentration: 10% (v/v) HFIP); and kept at room temperature under constant stirring. After 2 days, the tube was transferred to and maintained at 4°C.

### Thioflavin T (ThT) assay

Lyophilized Aβ40 peptide was first dissolved in HFIP on ice. Most of the HFIP was vacuum evaporated before Aβ was dissolved in H_2_O; PBS; or artificial cerebrospinal fluid (aCSF: 124 mM NaCl, 2.5 mM KCl, 2 mM CaCl_2_, 2 mM MgSO_4_, 1.25 mM NaH_2_PO_4_, 26 mM NaHCO_3_, and 10 mM glucose). Solubilized Aβ40 (5 µM) was mixed with a protein dissolved in a respective buffer. Note that different buffer conditions resulted in different aggregation kinetics of Aβ. ThT (10 µM unless otherwise noted) was added to each solution to assess the formation of β-sheet structures. Samples were incubated at 37°C in 96-well plates; triplicate measurements were taken every 30 min with an ARVO multilabel spectrofluorometer (PerkinElmer, USA). Plates were shaken for 10 sec prior to every measurement. Excitation and emission wavelengths were 440 and 486 nm, respectively [7]. In each experiment, triplicate wells containing protein sample at each concentration without Aβ were made. Results from the measurement of these wells were not included in the figures because the values were significantly smaller than those of wells with Aβ.

### Luciferase refolding assay

Twenty microliters of 250 nM luciferase (Sigma) in 100 mM Tris-HCl (pH 7.4) and 5 µL of 20 mM DTT in H_2_O were mixed in each well of a 96-well plate. For heat treatment, plates were sealed and placed in an incubator maintained at 45°C. After 5 min, the plates were taken out and kept at room temperature for 5 min. Next, 2.5 µL of 40 mM DTT (Nacalai Tesque), samples representing four different conditions, and 2.5 µL of 80 mM Mg-ATP (Sigma) in H_2_O were added to each well. The four samples were as follows: (1) 15 µL of H_2_O; (2) 5 µL of H_2_O, 5 µL of 12.5 µM GroEL, and 5 µL of 21 µM GroES; (3) 5 µL of 1 mg/mL A11, 5 µL of 12.5 µM GroEL, and 5 µL of 21 µM GroES; and (4) 5 µL of 1 mg/mL A11, and 10 µL of H_2_O. The plate was incubated at 30°C for 1 h. As soon as 5 µL of 2 mM luciferin (Nacalai Tesque) dissolved in 100 mM Tris-HCl (pH 7.4) was added to each well, the luminescence was measured with an ARVO multilabel spectrofluorometer (PerkinElmer). The average luminescence value from H_2_O-incubated wells was subtracted from the luminescence value measured from other wells. The percentage of luminescence recorded in each well was calculated, taking as 100% the average value measured from wells that were not heat treated. The percentages determined for each condition were averaged and defined as percent (%) luciferase activity. GroE represents the combination of both GroEL and GroES.

### Structural similarity search

Ten PDB files of A11-immunoreactive proteins were retrieved. In the preliminary search, 214 potential sites on the surfaces of ten structures were detected among the areas where the main β-sheet chain was exposed to the surface (Table 1). Atoms in these sites were first classified into six physicochemical types using the PATTY algorithm [50]. Structural alignment was carried out by aligning the spatial coordinates of atoms at every two sites and identifying pairs of atoms of the same physicochemical type. We then calculated similarity scores for these aligned structures. Twenty-three sites with relatively high similarity scores were determined using a clustering algorithm. For each structure, we selected sites having the highest similarity score. We then constructed a figure by performing multiple structure alignments on the ten sites having the highest similarity scores. Surface atoms were determined by calculating accessible surface areas (>0 Å^2^) at 1.4 Å of probe atoms. The atoms were clustered using the single-linkage clustering algorithm with a cutoff value of 4 Å. Clusters formed by less than four atoms were removed. Surface atoms that existed within 4 Å from atoms of the remaining clusters were included. Each cluster was defined as a single site.

**Table 1:** Potential surface sites on ten A11-reactive proteins in which the main β-sheet chain is exposed to the surface.

PDB code	Number of sites
1aon	114
1ayo	8
1c3g	6
1dkz	5
1dvd	7
1ikq	7
1mdt	15
1qvr	13
1yuw	13
2cg9	26
Total	214

#### Similarity score

Each atom located on a structure's surface was given a feature vector. The feature vector describes the local physicochemical environment of a surface atom. Atoms were first classified into six physicochemical types using the PATTY algorithm [50]: cation (*AT*1), anion (*AT*2), hydrogen-bond donor (*AT*3), hydrogen-bond acceptor (*AT*4), hydrophobic (*AT*5), and none of these (*AT*6). An atom that was both a hydrogen-bond donor and an acceptor (‘polar’ atom, according to the PATTY algorithm) was treated as 0.5 donor and 0.5 acceptor. The feature vectors were defined for each atom according to this atom-type classification. The feature vector **C** *_i_* of atom *i* was defined as:
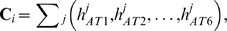
in which the summation was over all the solvent-accessible atoms (including atom *i*) of the protein, 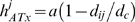 if *d_ij_*/*d_c_*≤1, and atom *j* is of type *ATx*; otherwise, 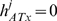. If atom *j* is both a donor and an acceptor and *x* = 3 or 4, *a* = 0.5; otherwise, a = 1. *d_ij_* is the distance (in Å) between atom *i* and atom *j*. The parameter values *d_c_* = 3.2 were used. The similarity between each pair of atoms from the two regions was assessed by *s_ij_* = *T_C_*(*i*, *j*), where atoms *i* and *j* were from regions *a* and *b*, respectively; and *T_C_*(*i*,*j*) is the Tanimoto coefficient:
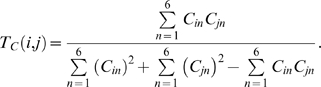



Finally, a similarity score *S* was defined for the two regions *a* and *b*: *S* = Σ*s_ij_*/min{*N_a_*,*N_b_*}, where *s_ij_* is defined as above; *N_a_* and *N_b_* are the numbers of atoms of regions *a* and *b*, respectively; and the summation was over all atom pairs in the alignment except for those pairs of different atom types. Possible *S* values ranged from 0 to 1, where 1 represents the maximum similarity.

## Supporting Information

Figure S1 Sites having the most similar physicochemical properties in the molecular structures of ten A11-reactive proteins. Because the aligned site in 1aon was hidden from view at the viewing angle we used for the other structures, only 1aon is shown from a different angle from other molecules. (A) GroEL [1aon]. (B) α2MG [1ayo]. (C) Hsp40 [1c3g]. (D) DnaK (Hsp70) [1dkz]. (E) TTR (prealbumin) [1dvq]. (F) ETA [1ikq]. (G) DT [1mdt]. (H) ClpB (Hsp104) [1qvr]. (I) Hsc70 [1yuw]. (J) Hsp90 [2cg9].
(8.92 MB TIF)
