# Supplementary figures and images for: Amyloid Oligomer Conformation in a Group of Natively Folded Proteins
Source: PLoS One. 2008 Sep 18;3(9):e3235. doi: 10.1371/journal.pone.0003235 (PMC2528939; doi:10.1371/journal.pone.0003235)

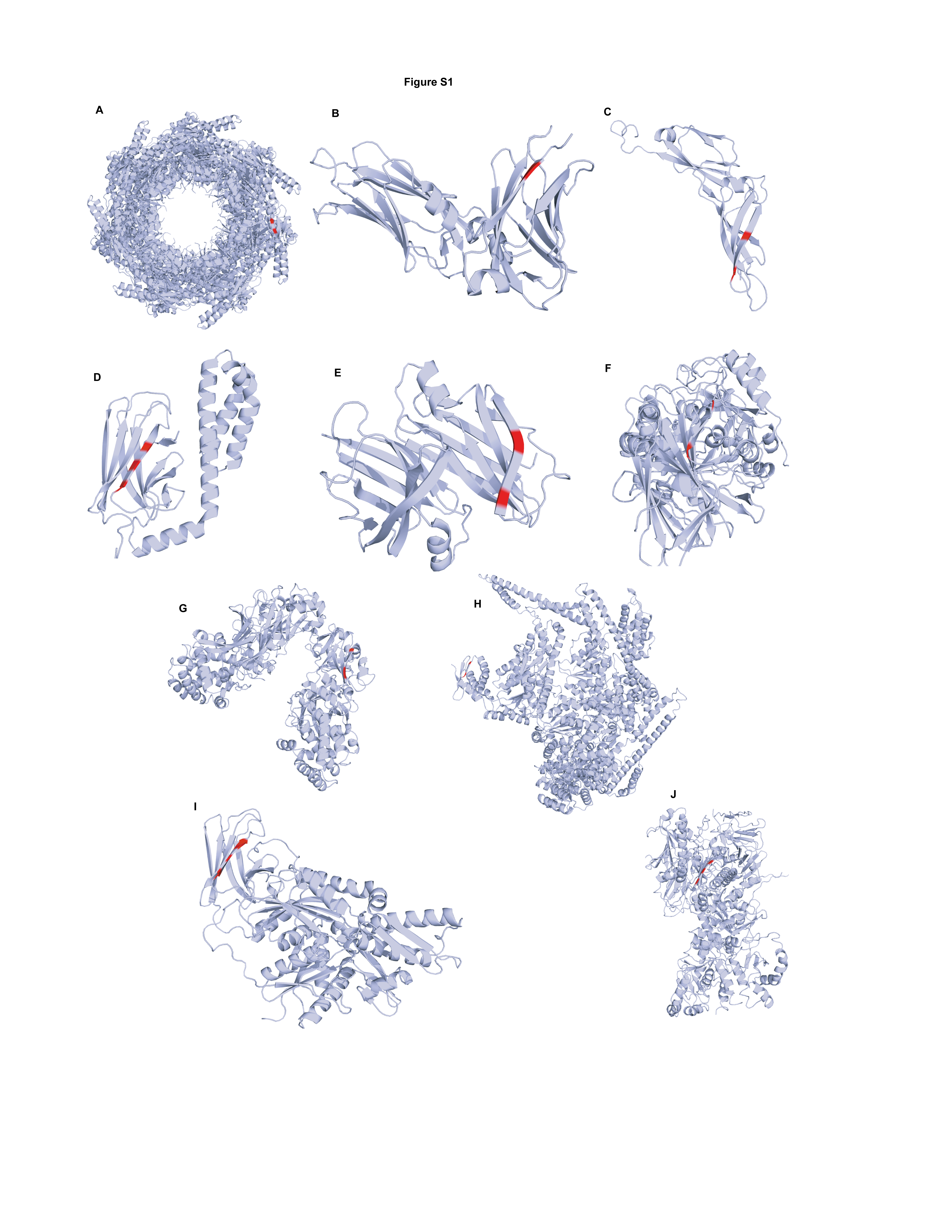

Supplement: Figure S1 — Sites having the most similar physicochemical properties in the molecular structures of ten A11-reactive proteins. Because the aligned site in 1aon was hidden from view at the viewing angle we used for the other structures, only 1aon is shown from a different angle from other molecules. (A) GroEL [1aon]. (B) α2MG [1ayo]. (C) Hsp40 [1c3g]. (D) DnaK (Hsp70) [1dkz]. (E) TTR (prealbumin) [1dvq]. (F) ETA [1ikq]. (G) DT [1mdt]. (H) ClpB (Hsp104) [1qvr]. (I) Hsc70 [1yuw]. (J) Hsp90 [2cg9]. (8.92 MB TIF) [file pone.0003235.s001.tif]
